# Effect of autophagy on aging-related changes in orthodontic tooth movement in rats

**DOI:** 10.1186/s12903-024-04549-3

**Published:** 2024-07-13

**Authors:** Bowen Xu, Chuhan Peng, Yugui Du, Qiuying Li, Kai Yang

**Affiliations:** Department of Orthodontics, School of Stomatology, Capital Medical University, Tiantan Xili No. 4, Dongcheng District, Beijing, China

**Keywords:** Tooth movement, Bone remodeling, Autophagy, Aging

## Abstract

**Background:**

The number of adult orthodontic patients is increasing, and studies have shown that autophagy is involved in regulating orthodontic tooth movement and plays an important role in aging-related changes. Therefore, we aimed to explore the role of autophagy in aging-related changes during orthodontic tooth movement by establishing a rat orthodontic tooth movement model.

**Methods:**

Forty-five 6-week-old and sixty-five 8-month-old male Sprague–Dawley rats were selected to represent adolescents and adults and establish orthodontic tooth movement model. They were sacrificed on days 0,1,3,7 and 14. Immunohistochemistry, immunofluorescence and tartrate resistant acid phosphatase (TRAP) staining were applied to measure the expression level of osteogenesis, autophagy, aging factors and osteoclast number in periodontal membrane of left upper first molar during orthodontic tooth movement. Then, we regulated the autophagy level by injecting autophagy activator rapamycin during orthodontic tooth movement and measured these factors and tooth movement distance by micro-computed tomography.

**Results:**

Aging factor levels in the periodontal membrane were higher in adult rats than in adolescent rats and the autophagy factor levels were lower. The levels of osteogenic factors were lower on the tension side in adult rats than in adolescent rats. The peak osteoclast number on the pressure side occurred later in adult rats than in adolescent rats. The injection of rapamycin increased autophagy, accelerated orthodontic tooth movement in adult rats, and reduced the levels of aging factors. The levels of osteogenic factors were higher and reached those in adolescent rats at some time points. The number of osteoclasts increased significantly in the early stage.

**Conclusions:**

Autophagy may play a substantial role in regulating aging-related changes in orthodontic tooth movement.

## Background

The key to effective bone remodeling during orthodontic tooth movement is a balance between osteoblast and osteoclast activity in alveolar bone, during which, stem cells in the periodontal membrane (periodontal ligament stem cells, PDLSCs) sense mechanical stimulation and participate in alveolar bone reconstruction. Our previous study showed that when cultured in vitro under 12% static tension, PDLSCs showed changes in the main markers of osteogenesis and autophagy. When regulated with drugs, increased autophagy levels could promote osteogenic differentiation, and vice versa [1]. Moreover, many studies have determined the positive effect of autophagy on osteoclast maturation, differentiation and migration [2, 3].

On the other hand, with the development of orthodontic technology, an increasing number of adults became willing to seek orthodontic treatment to improve their maxillofacial aesthetics[4]. It was clear that the course of treatment for adult patients was much longer than that for adolescents. We compared the features of bone remodeling during orthodontic treatment between adult and adolescent rats and found that the tooth movement speed in adult rats was slower [5]. However, the reason behind remains unknown. Kapoor et al. explored the levels of cytokines, chemokines, their associated receptors and antagonists in gingival crevicular fluid (GCF) under orthodontic force in a systematic review and concluded that the ratio of receptor activator of nuclear factor kappa-B ligand(RANKL) to osteoprotegerin(OPG) (RANKL/OPG) and activity index (AI) (interleukin-1β/interleukin-1RA, IL-1β/IL-1RA) were higher in GCF during OTM in adolescents compared to adults [6]. Zhang et al. cultured human periodontal ligament stem cells (hPDLSCs) from subjects in different age groups and found that as age increased, the proliferation, migratory potential and differentiation capacity of hPDLSCs correspondingly decreased, indicating aging-related changes in hPDLSCs [7]. Some studies have revealed the vital effect of autophagy on aging-related changes in stem cells. Ma et al. demonstrated that autophagy activity decreased with age in bone marrow-derived mesenchymal stem cells (BMMSCs) compared with that of cells derived from 3- and 16-month-old mice. The autophagy inhibitor 3-methyladenine (3 - MA) could turn young BMMSCs into a relatively aged state by reducing their osteogenic differentiation, and the autophagy activator rapamycin could restore the capacity for osteogenic differentiation in cells from aged mice [8]. Thus, autophagy is significant in aging-related changes for stem cells and may play an important role in aging-related changes in orthodontic tooth movement.

Therefore, in this research, we aimed to explore the relationship between autophagy and aging-related changes in orthodontic tooth movement to provide new ideas for improving the efficiency of orthodontic treatment, especially for adult patients.

## Methods

### Animals

One hundred and ten male Sprague-Dawley (SD) rats (SPF Biotechnology, Beijing, China) were used. 45 rats were 6 weeks old and 65 were 8 months old to represent the adolescent and adult groups respectively. They were housed in the animal experiment center of Beijing Stomatological Hospital under a relatively stable temperature (22 ± 2.0℃) and a 12-hour light/dark cycle. All animal experimental procedures comply with the ARRIVE guidelines and this research was approved by the Animal Ethics and Welfare Committee of the School of Stomatology, Capital Medical University (No. KQYY-202207-005).

### Establishment of orthodontic tooth movement model and injection of drugs

**Fig. 1:**
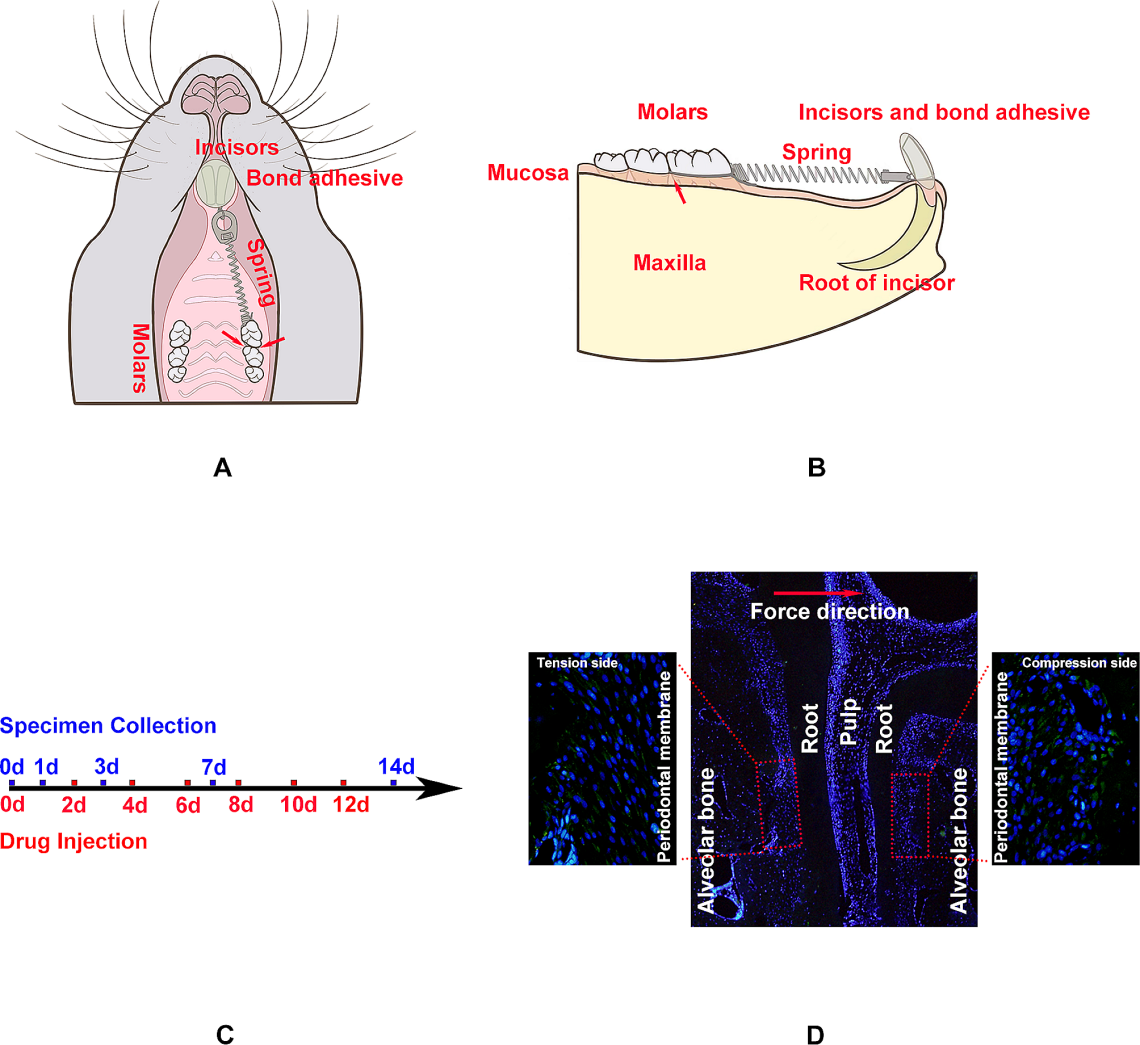
Illustration of the experimental methods **A, B** Diagram of orthodontic tooth movement model establishment and drug injection. Arrows show the drug injection points **C** Flowchart of drug injection and specimen collection **D** Diagram of the zone of interest for histopathological analysis on the tension side and compression side of the periodontal ligament of the left maxillary first molar

After adaptive feeding for one week, the rats were subjected to orthodontic tooth movement after anesthesia by an intraperitoneal injection of 1.25% tribromoethanol (Avertin, 10 ml/kg, M2960, AibeiBio, Nanjing, China). The maxillary left first molar was chosen as an experimental tooth and incisor as an anchor tooth. We used a nickel-titanium (Ni-Ti) coil spring and ligature wire to connect them and subjected the experimental tooth to 25 g of pull force. Bond adhesive was used to help fix the device (Fig. 1A, B). A soft diet was provided to avoid displacement and breakage of the device.

Rapamycin, also known as sirolimus, is a macrolide antibiotic that inhibits the mTOR signaling pathway to induce autophagy [9]. In adult rats, during orthodontic force loading, rapamycin (10 µg/mL, Solarbio, Beijing, China) was injected into the buccal and palatal mucosa of the left maxilla first molar distal buccal root at a dose of 200 µL on each side. Normal saline (SJZ NO.4 Pharmaceutical, Hebei, China) was injected at the same points and same dose in both adult and adolescent rats as controls. Both normal saline and rapamycin were injected once every other day (Fig. 1C).

### Specimen collection

Before force loading, 5 adolescent and 5 adult rats were selected randomly and sacrificed by cervical dislocation to set a baseline. Other rats were divided into different groups after orthodontic force loading: 20 adult rats and 20 adolescent rats not administered any injections (adult and adolescent); 20 adult rats and 20 adolescent rats injected with normal saline (saline-adult and saline- adolescent); and 20 adult rats injected with rapamycin (rapa-adult). They were sacrificed at different time points after orthodontic force loading (days 1, 3, 7, and 14, *n* = 5). All specimens were fixed in 4% paraformaldehyde fixation solution for 24 h and then washed in phosphate buffered saline (PBS) once a day for three days.

### Tooth movement distance measurement

**Fig. 2:**
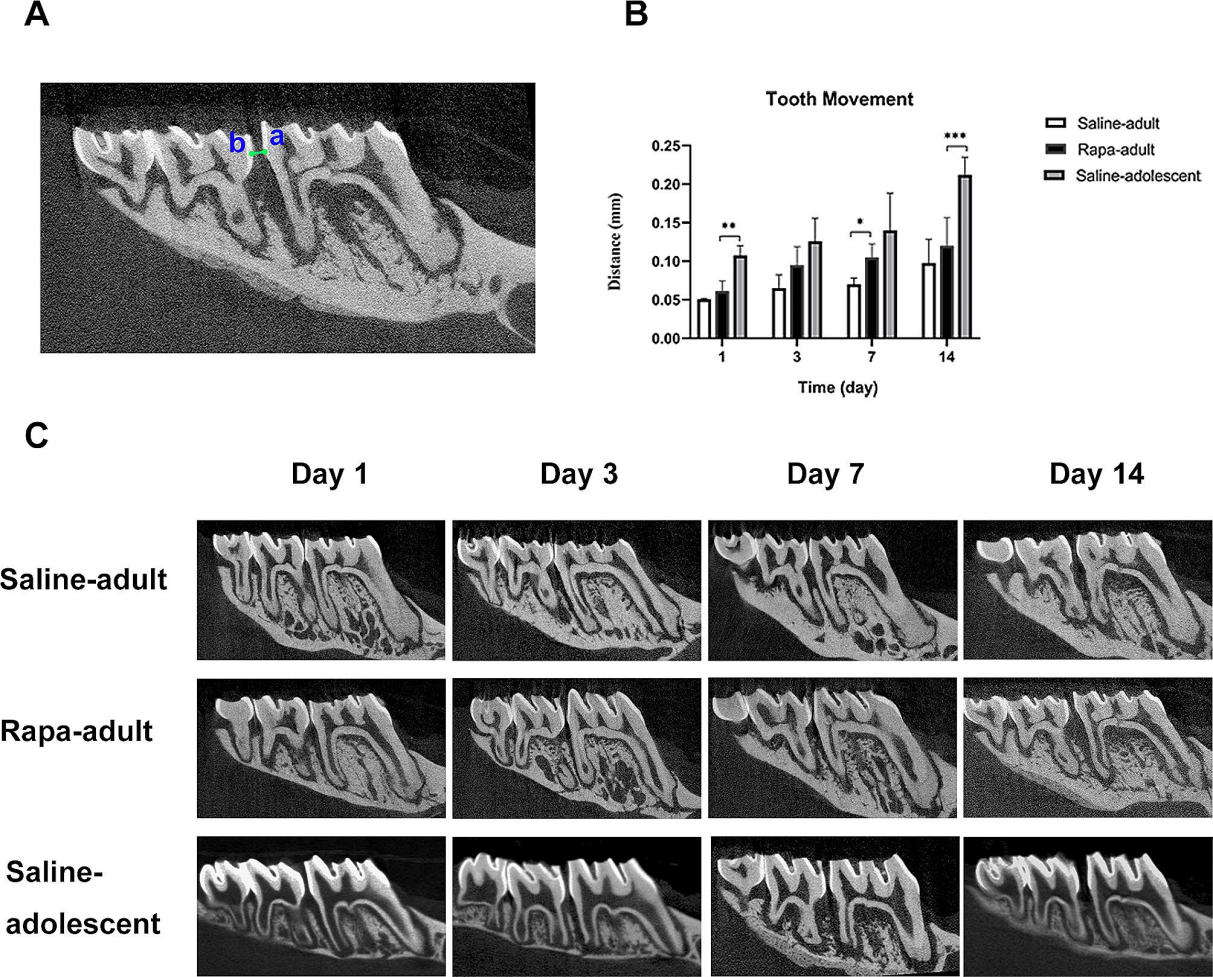
Orthodontic tooth movement in saline-adult, rapa-adult and saline-adolescent rats **A** Method of orthodontic tooth movement evaluation. Point a refers to the most distal point of the crown of the maxillary left first molar, and point b refers to the most mesial point of the crown of the second molar. The distance of line ab represents the orthodontic tooth movement distance **B** Comparison of the orthodontic tooth movement distance between the saline-adult, rapa-adult and saline-adolescent rats. (*n* = 5, *P < 0.05, **P < 0.01, ***P < 0.001) **C** Comparison of orthodontic tooth movement between the saline-adult, rapa-adult and saline-adolescent rats on micro-computed tomography

To measure the orthodontic tooth movement distance, we scanned each sample by SkyScan1276 (SkyScan, Kontich, Belgium) and reconstructed the data. A total of 1383 slices from each sample were collected in BMP format, with a pixel size of 8.04206 μm. DataViewer was used to correct the direction on each axis and measure the orthodontic tooth movement distance, which was defined as the distance between the distal convex point of the left maxillary first molar crown and the mesial convex point of the second molar crown (Fig. 2A).

### Histopathological analysis

Specimens were soaked in 10% EDTA (BL617A, Biosharp, Anhui, China) for approximately 60 days for decalcification, dehydrated by a series of different alcohol concentrations and then embedded in paraffin according to a previously described conventional method. Serial 5 μm sections were cut for histopathological analysis.

The samples were stained for expression of the osteogenesis markers Runx-2 (1:1000, ab192256, Abcam, Cambridge, England) and Osterix (1:1000, ab209484, Abcam, Cambridge, England) by immunohistochemistry. These proteins, present in the nuclei, appear brown when osteogenesis is active. The other nuclei were stained blue by hematoxylin. The percentage of positive cells in the periodontal membrane of the first molar on the tension side represented the level of osteogenic activity. Mature osteoclasts could be stained by tartrate resistant acid phosphatase (TRAP) (G1492, Solarbio, Beijing, China), appearing red, with multiple nuclei. The number of mature osteoclasts on the compression side indicated the level of osteoclast activity.

Sections were subjected to immunofluorescence staining with a conventional procedure to calculate the average fluorescence intensity as a representation of the levels of autophagy and aging markers on both the tension and compression sides. Antibodies against the autophagy markers LC3B (1:500, ab48394, Abcam, Cambridge, England) and ATG7 (1:500, ab133528, Abcam, Cambridge, England) and the aging marker P16 (ab51243, Abcam, Cambridge, England) were selected. Nuclei were stained with 4’,6-diamidino-2-phenylindole (DAPI) (F6057, Sigma-Aldrich, USA). Fluorescence microscope was applied to observe the zone of interest, and Image-Pro Plus 6.0 was used to calculate the fluorescence intensity (Fig. 1D).

### Statistical analysis

All data were recorded and analyzed by IBM SPSS Statistics 23 (IBM, USA) software. An independent samples t-test was used to analyze the differences of the expression levels of LC3, ATG7, P16, Runx-2, Osterix, osteoclast number and the tooth movement distance at each time point between group adult and adolescent; rapa-adult and saline-adult; rapa-adult and saline-adolescent. *P* < 0.05 was considered significant.

## Results

### Autophagy, aging and osteogenesis factors in the periodontal membrane on the tension side in adolescent and adult rats

**Fig. 3:**
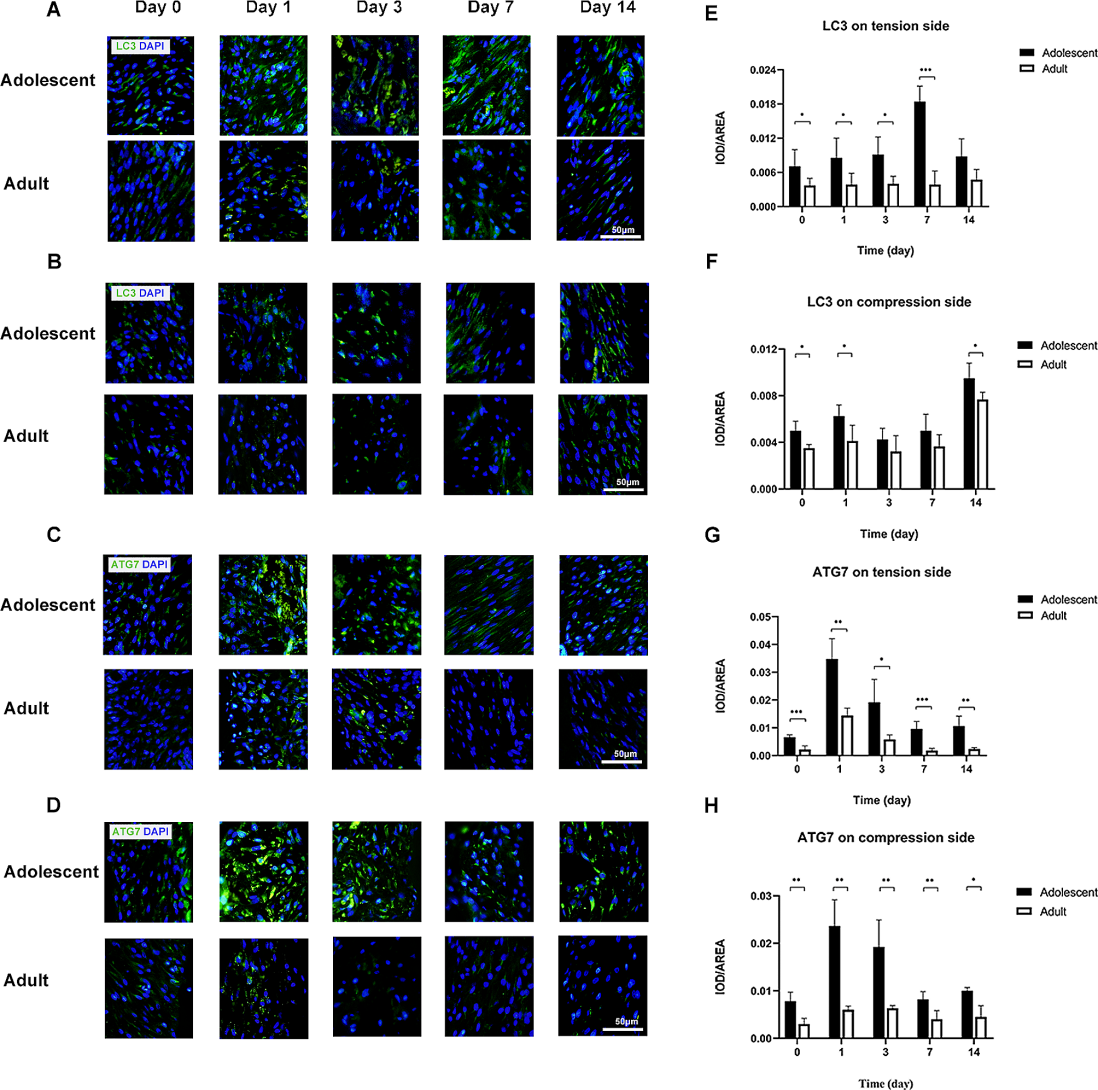
Autophagy level on tension and compression side of periodontal membrane in adolescent and adult rats **A** LC3 in the periodontal membrane on the tension side in adolescent and adult rats by immunofluorescence staining, with LC3 in green and nuclei in blue **B** LC3 in the periodontal membrane on the compression side in adolescent and adult rats by immunofluorescence staining, with LC3 in green and nuclei in blue. **C** ATG7 in the periodontal membrane on the tension side in adolescent and adult rats by immunofluorescence staining, with ATG7 in green and nuclei in blue **D** ATG7 in the periodontal membrane on the compression side in adolescent and adult rats by immunofluorescence staining, with ATG7 in green and nuclei in blue. Scale bar indicates 50 μm **E** Expression level of LC3 in the periodontal membrane on the tension side in adolescent and adult rats **F** Expression level of LC3 in the periodontal membrane on the com-pression side in adolescent and adult rats **G** Expression level of ATG7 in the periodontal membrane on the tension side in adolescent and adult rats **H** Expression level of ATG7 in the periodontal membrane on the compression side in adolescent and adult rats. (*n* = 5, *P < 0.05, **P < 0.01, ***P < 0.001)

The levels of the autophagy markers LC3 and ATG7 on the tension side in adult rats were lower than those in adolescent rats before force loading. With the application of orthodontic force, the expression level of LC3 in adolescent rats increased to the peak on day 7 and then returned to the initial level. The level in adult rats was significantly lower than that of adolescent rats at all time points (Fig. 3A, E). The level of ATG7 in adolescent rats peaked on day 1 and then returned to the initial level and was higher than that in adult rats at all time points (Fig. 3C, G).

**Fig. 4:**
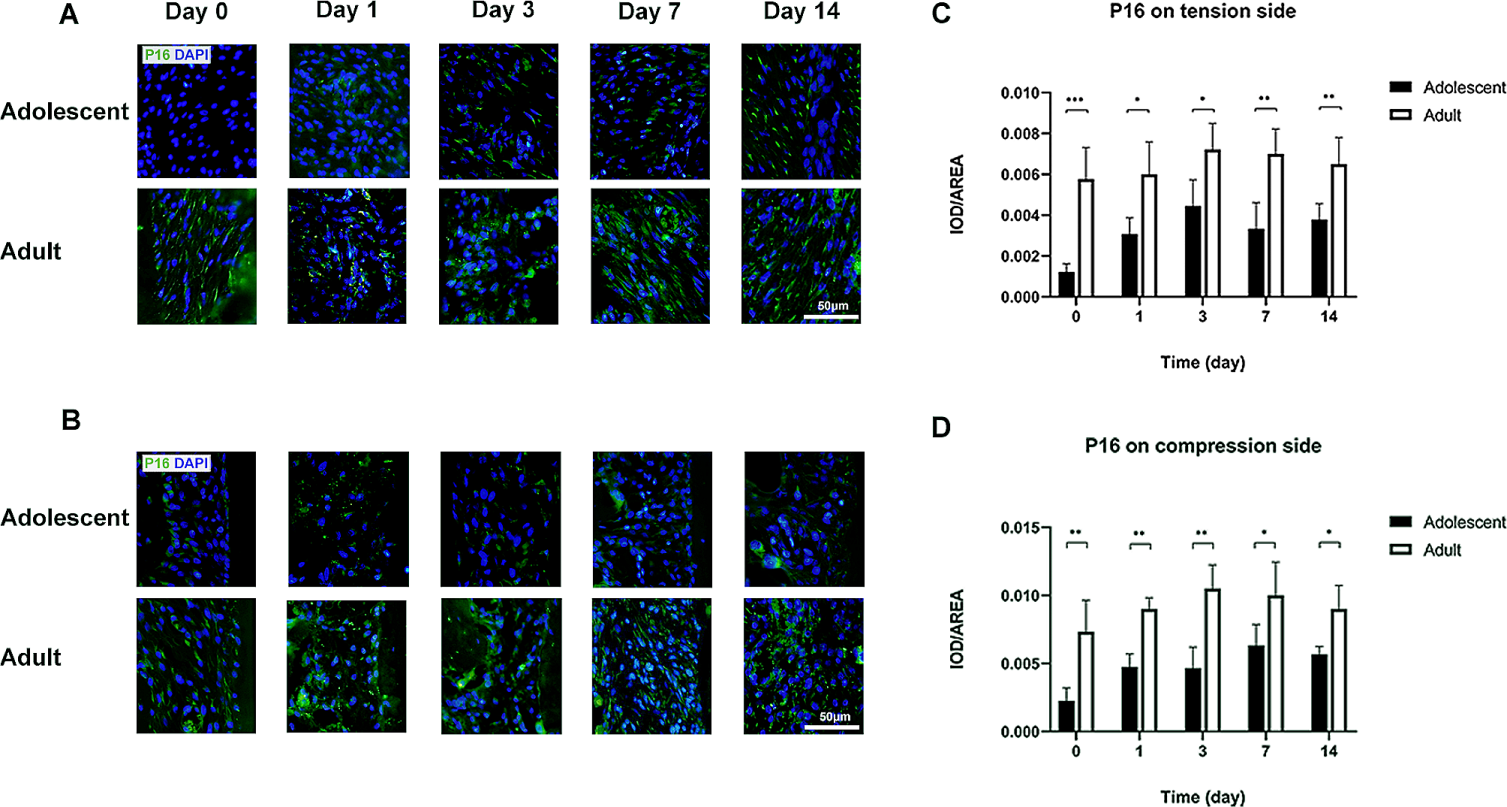
P16 level on tension and compression side of periodontal membrane in adolescent and adult rats **A** P16 in the periodontal membrane on the tension side in adolescent and adult rats by immunofluorescence staining, with P16 in green and nuclei in blue **B** P16 in the periodontal membrane on the compression side between adolescent and adult rats by immunofluorescence staining, with P16 in green and nuclei in blue. Scale bar indicates 50 μm **C** Expression level of P16 in the periodontal membrane on the tension side in adolescent and adult rats **D** Expression level of P16 in the periodontal membrane on the compression side in adolescent and adult rats. (*n* = 5, *P < 0.05, **P < 0.01, ***P < 0.001)

The level of the aging factor P16 on the tension side in adult rats was significantly higher than that in adolescent rats at all time points (Fig. 4A, C).

**Fig. 5:**
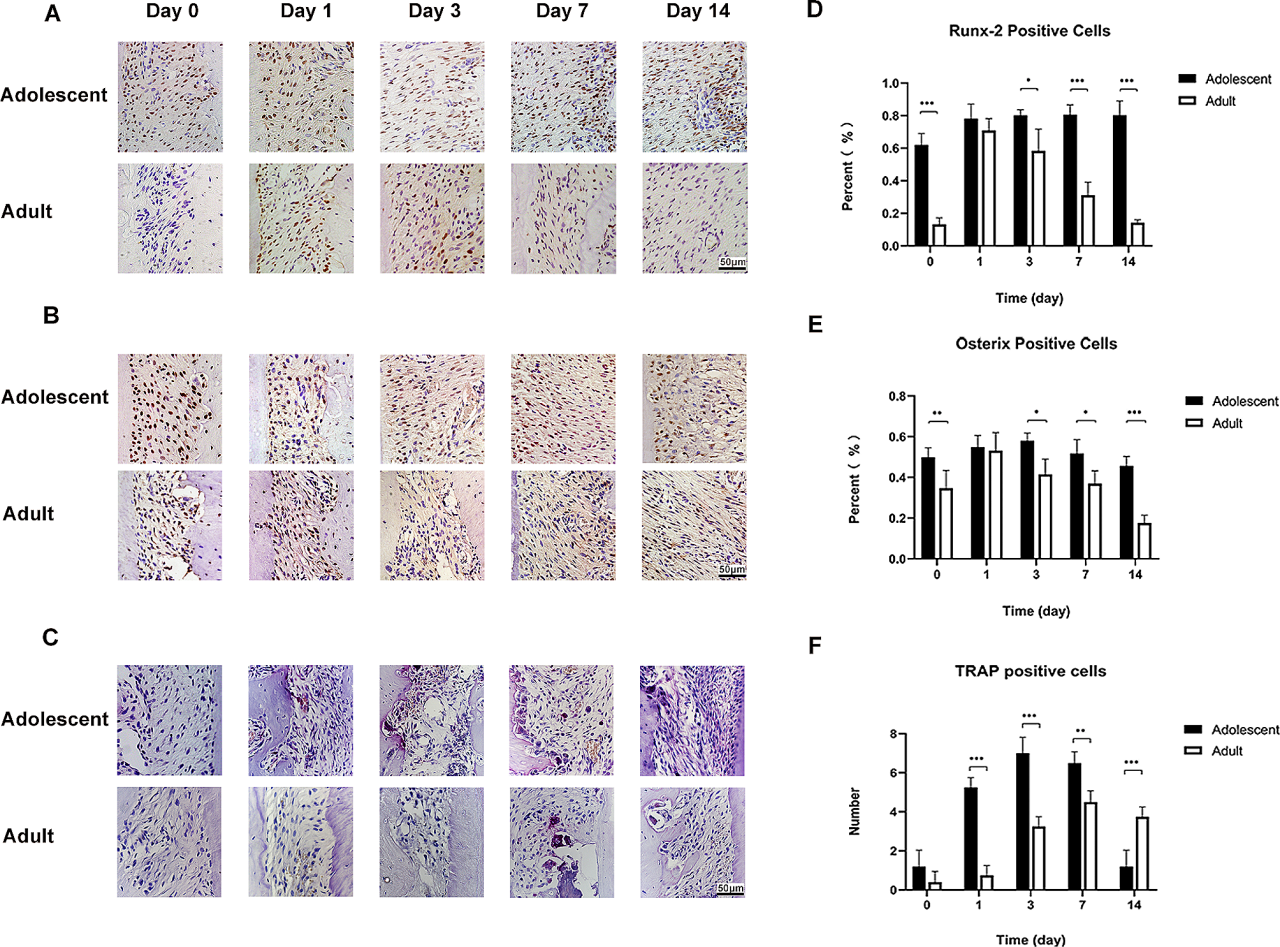
Osteogenesis level and osteoclast number in periodontal membrane in adolescent and adult rats **A** Runx-2-positive cells in the periodontal membrane on the tension side in adolescent and adult rats by immunohistochemistry **B** Osterix-positive cells in the periodontal membrane on the tension side in adolescent and adult rats by immunohistochemistry **C** Mature osteoclasts stained by TRAP (red, with multiple nuclei) in the periodontal membrane on the compression side in adolescent and adult rats. Scale bar indicates 50 μm **D** Percentage of Runx-2-positive cells in the periodontal membrane on the tension side in adolescent and adult rats **E** Percentage of Osterix-positive cells in the periodontal membrane on the tension side in adolescent and adult rats **F** Number of mature osteoclasts in the periodontal membrane on the compression side in adolescent and adult rats. (*n* = 5, *P < 0.05, **P < 0.01, ***P < 0.001)

Levels of the osteogenesis factors Runx-2 and Osterix were lower in adult rats than in adolescent rats before force loading. With the application of force, the Runx-2 expression level remained high in adolescent rats; in adult rats, it was highest on day 1 and decreased gradually to the initial level and was lower than that in adolescent rats on days 3, 7 and 14 (Fig. 5A, D). The Osterix level in both adult and adolescent rats tended to increase first and then decrease; on days 3, 7 and 14, it was significantly lower in adult rats than in adolescent rats (Fig. 5B, E).

### Autophagy, aging factors and mature osteoclasts in the periodontal membrane on the compression side in adolescent and adult rats

The levels of both LC3 and ATG7 were lower in adult rats than in adolescent rats before orthodontic treatment. With the application of force, the LC3 level was lowest on day 3 and increased to the highest level on day 14 in both groups. The LC3 level was significantly lower in adult rats than in adolescent rats at each time point (Fig. 3B, F). The ATG7 level peaked on day 1 and then returned to the original level in adolescent rats; in adult rats, it showed little change and was always lower than that in adolescent rats (Fig. 3D, H).

The P16 level on the compression side was significantly higher in adult rats at each time point than in adolescent rats (Fig. 4B, D).

The number of mature osteoclasts peaked on day 3 in adolescent rats and on day 7 in adult rats, at a lower level. The number of osteoclasts in adult rats was lower than that in adolescent rats on days 1, 3, 7 and 14. (Fig. 5C, F).

### Orthodontic tooth movement in rapa-adult, saline-adult and saline-adolescent rats

The orthodontic tooth movement distance in all groups increased with time. Distance in rapa-adult rats was longer than that in saline-adult rats, especially on day 7. The distance in rapa-adult rats was lower than that in saline-adolescent rats at all time points. (Fig. 2B, C).

### Autophagy and aging factors in the periodontal membrane on the tension side in rapa-adult and saline-adult rats

**Fig. 6:**
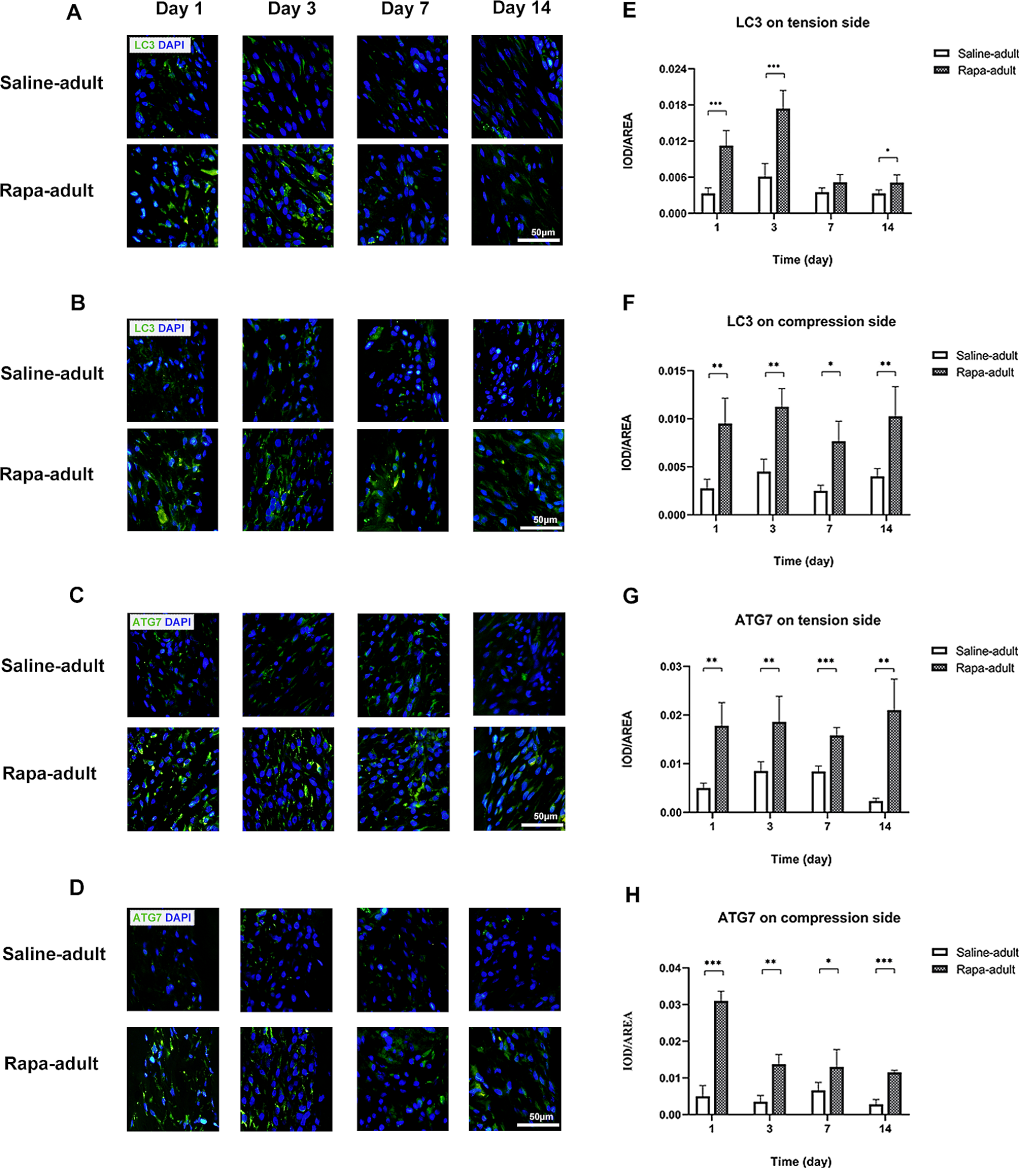
Autophagy level on tension and compression side of periodontal membrane in saline-adult and rapa-adult rats **A** LC3 in the periodontal membrane on the tension side in saline-adult and rapa-adult rats by immunofluorescence staining, with LC3 in green and nuclei in blue **B** LC3 in the periodontal membrane on the compression side in saline-adult and rapa-adult rats by immunofluorescence staining, with LC3 in green and nuclei in blue **C** ATG7 in the periodontal membrane on the tension side in saline-adult and rapa-adult rats by immunofluorescence staining, with in ATG7 green and nuclei in blue **D** ATG7 in the periodontal membrane on the compression side in saline-adult and rapa-adult rats by immunofluorescence staining, with ATG7 in green and nuclei in blue. Scale bar indicates 50 μm **E** Expression level of LC3 in the periodontal membrane on the tension side in saline-adult and rapa-adult rats **F** Expression level of LC3 in the periodontal membrane on the compression side in saline-adult and rapa-adult rats **G** Expression level of ATG7 in the periodontal membrane on the tension side in saline-adult and rapa-adult rats **H** Expression level of ATG7 in the periodontal membrane on the compression side in saline-adult and rapa-adult rats. (*n* = 5, *P < 0.05, **P < 0.01, ***P < 0.001)

The expression level of the autophagy marker LC3 on the tension side was higher in rapa-adult rats than in saline-adult rats at all time points, with a significant difference on days 1, 3 and 14 (Fig. 6A, E). The ATG7 level was higher in rapa-adult rats than saline-adult rats at all time points (Fig. 6C, G).

**Fig. 7:**
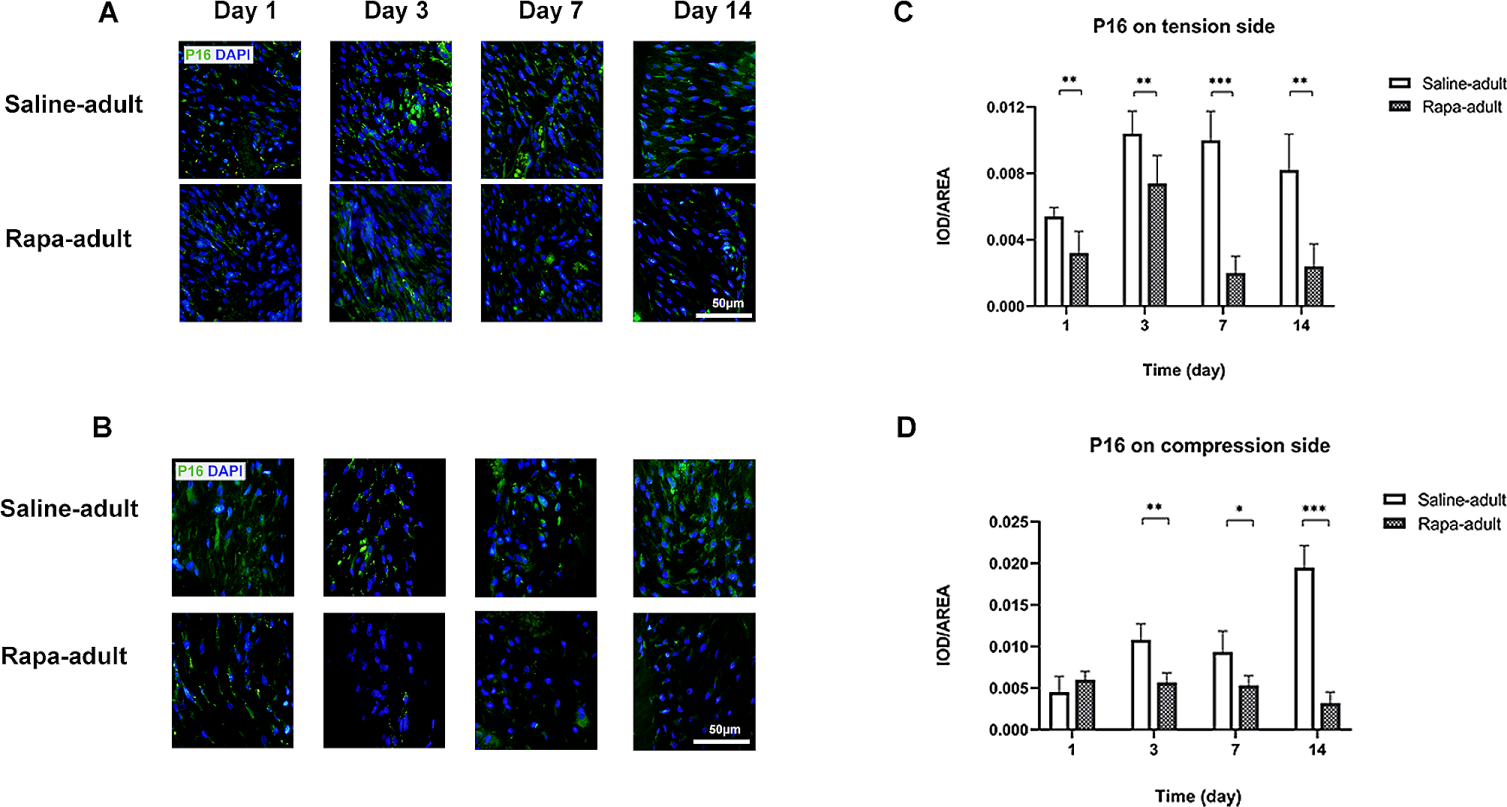
P16 level on tension and compression side of periodontal membrane in saline-adult and rapa-adult rats **A** P16 in the periodontal membrane on the tension side in saline-adult and rapa-adult rats by immunofluorescence staining, with P16 in green and nuclei in blue. **B** P16 in the periodontal membrane on the compression side in saline-adult and rapa-adult rats by immunofluorescence staining, with P16 in green and nuclei in blue. Scale bar indicates 50 μm **C** Expression level of P16 in the periodontal membrane on the tension side in saline-adult and rapa-adult rats **D** Expression level of P16 in the periodontal membrane on the compression side in saline-adult and rapa-adult rats. (*n* = 5, *P < 0.05, **P < 0.01, ***P < 0.001)

The expression level of the aging factor P16 on the tension side was significantly lower in rapa-adult rats than in saline-adult rats at all time points (Fig. 7A, C).

### Autophagy and aging factors in the periodontal membrane on the compression side in rapa-adult and saline-adult rats

The levels of the autophagy markers LC3 and ATG7 on the compression side were significantly higher in rapa-adult rats than in saline-adult rats at all time points (Fig. 6B, D, F and H).

The level of aging factor P16 on the compression side in rapa-adult rats was significantly lower than that in saline-adult rats on days 3, 7 and 14 (Fig. 7B, D).

### Osteogenesis factors in the periodontal membrane on the tension side in rapa-adult, saline-adult and saline-adolescent rats

**Fig. 8:**
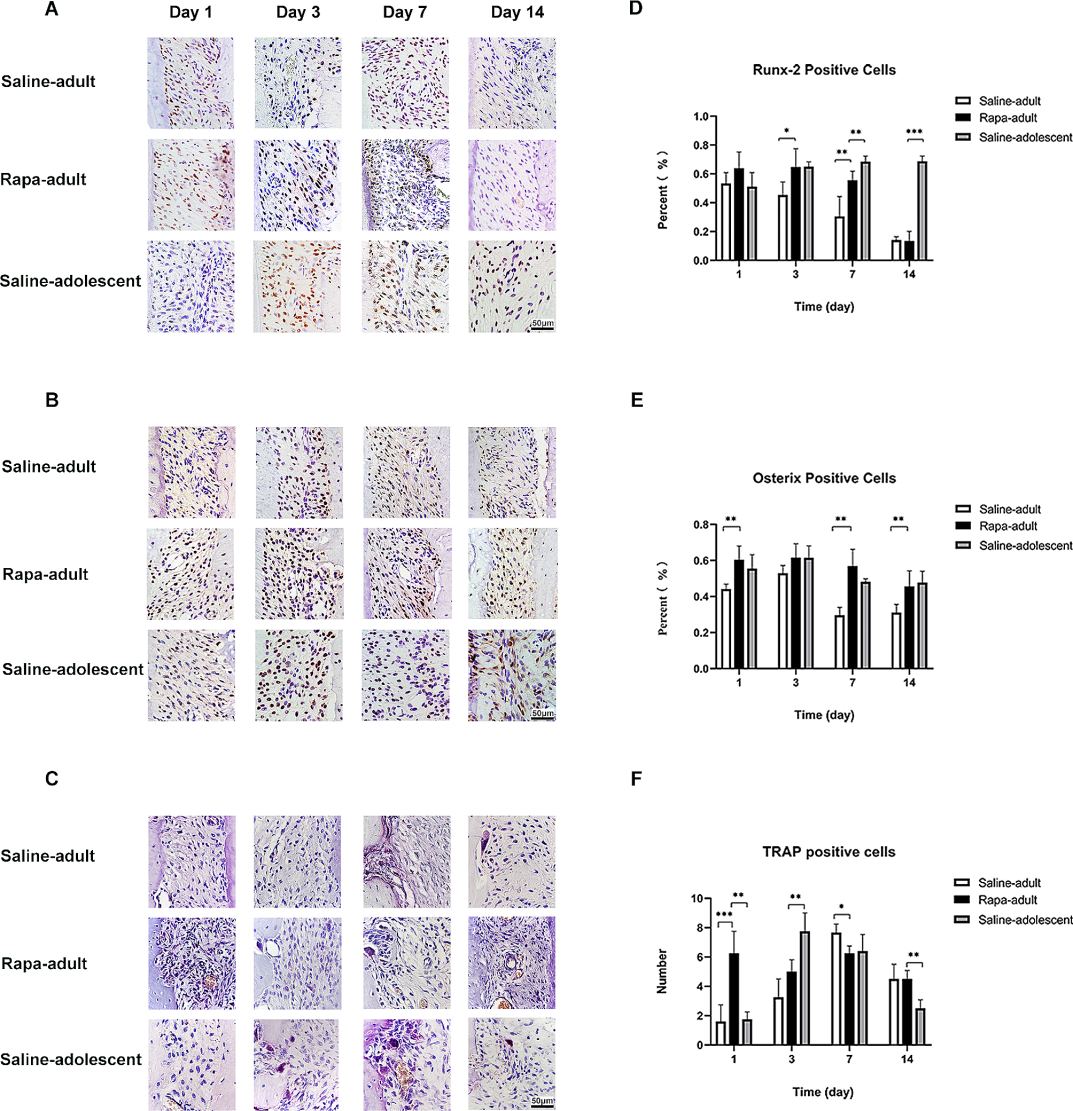
Osteogenesis level and osteoclast number in periodontal membrane in saline-adult, rapa-adult and saline-adolescent rats **A** Runx-2-positive cells in the periodontal membrane on the tension side in saline-adult, rapa-adult and saline-adolescent rats by immunohistochemistry **B** Osterix-positive cells in the periodontal membrane on the tension side in saline-adult, rapa-adult and saline-adolescent rats by immunohistochemistry **C** Mature osteoclasts stained by TRAP (red, with multiple nuclei) in the periodontal membrane on the compression side in saline-adult, rapa-adult and saline-adolescent rats. Scale bar indicates 50 μm **D** Percentage of Runx-2 positive cells in the periodontal membrane on the tension side in saline-adult, rapa-adult and saline-adolescent rats **E** Percentage of Osterix-positive cells in the periodontal membrane on the tension side in saline-adult, rapa-adult and saline-adolescent rats **F** Number of mature osteoclasts in the periodontal membrane on the compression side in saline-adult, rapa-adult and saline-adolescent rats. (*n* = 5, *P < 0.05, **P < 0.01, ***P < 0.001)

The percentage of Runx-2-positive cells was higher in rapa-adult rats than in saline-adult rats on days 3 and 7 with statistical difference. Moreover, the percentage of Osterix positive cells was higher in rapa-adult rats on days 1, 7 and 14 with statistical difference. With the increase in autophagy, Runx-2 expression on the tension side in rapa-adult rats reached the level in saline-adolescent rats on days 1 and 3, and Osterix expression reached the level in saline-adolescent rats on days 1, 3 and 7 (Fig. 8A, B, D and E).

### Mature osteoclasts in the periodontal membrane on the compression side in rapa-adult, saline-adult and saline-adolescent rats

The number of mature osteoclast on the compression side in saline-adult rats peaked on day 7. However, it was significantly higher in rapa-adult rats on the first day than in saline-adult rats and then remained high. On days 1 and 14, the number was even higher than that in saline-adolescent rats with statistical difference (Fig. 8C, F).

## Discussion

P16, a cyclin-dependent kinase inhibitor, tumor inhibitor, and aging-related marker, is often used as a relatively mature and stable detection marker in physiological aging and aging-related disease research [10]. In this research, we demonstrated that the level of P16 was higher in adult rats on both the tension and compression sides, indicating an obvious aging-related change in the periodontal membrane. On the other hand, the expression levels of Runx-2 and Osterix were relatively low; the peak osteoclast number appeared late in adult rats, which illustrated less bone remodeling activity than in adolescent rats and was consistent with the aging factor changes in the periodontal membrane. This result is consistent with most research focusing on aging-related changes in orthodontic tooth movement [11–13, 31].

LC3, a soluble protein of approximately 17 kDa, has been commonly detected in mammalian tissues and cells. It is a well-recognized and reliable protein marker for evaluating autophagy level [14]. ATG7, a ubiquitin E1-like ligase, is present in both sets of ubiquitin-like systems in autophagy activity [15]. Therefore, we selected these two proteins to measure the level of autophagy in this study.

In adult rats, the expression levels of LC3 and ATG7 on both the tension and compression sides of the periodontal membrane were relatively low. With the application of orthodontic force, they fluctuated within a narrow range in adult rats and were always lower than those in adolescent rats. Therefore, we demonstrated a relationship between low autophagy levels and the aging-related changes during orthodontic tooth movement. With aging, the level of autophagy decreased in the periodontal ligament, which corresponds with the results of some previous studies. Couve et al. compared the autophagy activity of odontoblasts from young and adult individuals and found reduced autophagic activity in adult odontoblasts [16]. In addition, Wang et al. demonstrated that ATG7 regulated the senescence and proliferation of hPDLSCs [32].

To deeply explore the role of autophagy in aging-related changes, we increased the autophagy level in adult rats by injecting rapamycin. With the increase in autophagy by rapamycin, the level of the aging factor P16 decreased, while the levels of the osteogenesis factors Runx-2 and Osterix and the number of osteoclasts increased, indicating more active bone remodeling. This means that with the activation of autophagy, the level of aging factors of the periodontal membrane recovered to some degree, bone remodeling activity increased and then orthodontic tooth movement accelerated in adult rats.

We also compared the data between adult rats injected with rapamycin and adolescent rats injected with saline to confirm the positive role of autophagy in reversing the aging state. The results showed that factors involved in osteogenesis and the number of osteoclasts changed significantly in response to rapamycin-mediated regulation and even reached the levels in adolescent rats. This explains the vital role of autophagy in reversing aging-related changes to some degree. It agrees with the study by Ma et al. on the effect of autophagy on controlling aging-related changes in BMMSCs mentioned previously [8]. There are emerging reports on the function of autophagy in regulating aging-related changes. It has been reported that autophagy activity is significant in preventing the senescence of stem cells in mice [17] and that the activation of autophagy can restore the regenerative capacity in aging hematopoietic stem cells [18].

However, the exact mechanism and pathway of autophagy regulating aging-related changes remained unknown. Cordeiro et al. have found that rutin can enhance autophagy activity and delay aging stages and aging-related diseases through insulin/insulin-like growth factor-1 signaling pathway [19]. Sirtuin-1, a silence factor in mammals, may associate with the deacetylation of autophagy related proteins ATG5, ATG7, ATG8 and was considered to be a key factor in aging [20]. In addition, Tan et al. found that autophagy may control the aging process through PI3K/AKT/mTOR pathway [21].

In fact, researchers have never stopped exploring methods to accelerate OTM. Our study has identified a promising way to regulate OTM by manipulating autophagy levels, especially in adult patients. Some non-surgical methods of accelerating OTM have attracted the attention of orthodontics, such as low-intensity pulsed ultrasound (LIPUS), low-level laser therapy (LLLT), and vibrating appliances (e.g., AcceleDent and Tooth masseuse). LIPUS has been shown to be effective in accelerating OTM, reducing alveolar bone resorption and protecting the regenerative potential of bone tissue [22–26, 33]. LLLT has been reported to accelerate OTM by up to 34% or more [27, 28]. Supplementary vibration at low magnitude and high frequency was considered useful to accelerate OTM and some found its role in increasing transforming growth factor-β1 in osteocytes [29, 30]. However, the potential mechanism of these methods in accelerating OTM, especially in adults, needs further research.

In this research, we only focused on changes of protein expression in periodontal membrane and tooth movement in rats during orthodontic treatment. Further exploration is needed to understand the specific mechanisms and pathways by which autophagy regulates aging-related changes.

## Conclusions

In this study, we compared bone remodeling between adult and adolescent rats and found evidence of aging-related changes in bone remodeling and a decrease in the autophagy level with age during orthodontic tooth movement. Regulating the autophagy level of adult rats, the aging-related change was relieved to some degree, the bone remodeling capacity was increased, and the speed of orthodontic tooth movement also increased to some degree. These findings demonstrate that autophagy plays a substantial role in regulating aging-related changes during orthodontic tooth movement and may lead to new ideas to accelerate orthodontic tooth movement for patients, especially adult ones.

## Data Availability

The datasets used and analysed during the current study are available from the corresponding author on reasonable request.
